# Intelligent system for portfolio optimization for novel volatility forecasting using machine learning

**DOI:** 10.1038/s41598-026-42813-4

**Published:** 2026-05-08

**Authors:** Tamal Biswas, Animesh Dey, Gouranga Mandal, Neelanjan Ghosh

**Affiliations:** 1 School of Engineering & Architecture, Techno India University Tripura, Agartala, 799004 India; 2 Manipal Institute of Technology, Manipal Academy of Higher Education, Manipal, India; 3 Google LLC, Austin, TX USA

**Keywords:** Portfolio optimization, Multi-SVM classifier, Financial volatility, Risk management, Machine learning in finance, International portfolio, Engineering, Mathematics and computing

## Abstract

The study of portfolio optimization has been dedicated to a significant amount of literature because of its critical practical importance in the sphere of portfolio investment. The various theoretical frameworks and methodological constructs expressed by scholars have been aimed at optimizing portfolios to a better degree, i.e., reducing risk at the expense of increasing returns. The current study introduces a systematic machine-learning model of portfolio optimization that involves the combination of recognized analytical techniques. The sensitivity of the drawdown ratio of 40 countries is examined in terms of a multi-SVM decision-making representation. The system of classification was very accurate with a rate of 97.5%. The suggested solution contains analytical modelling and controlled data-preprocessing stages that guarantee the consistency of feature recovery and normalization of massive international-oriented datasets. The system has a high level of flexibility to the dynamism of the market because it uses a wide range of financial indicators and volatility-based parameters. Further, the multi-SVM ensemble is based on nonlinear kernel mappings to reveal the complex relationships between the risk and return components that make the process offer more accurate portfolio rebalancing information. Relative investigations with the traditional frameworks affirm better stability, higher risk management, and better precision in forecasting. The empirical analysis is also put in perspective with respect to the recent machine learning and deep reinforcement learning-based frameworks of portfolio optimization published in the recent literature. Lastly, this framework provides a scalable basis of integration with reinforcement learning and hybrid AI-based decision systems, thus creating consistent and data-driven financial approaches to global portfolio management.

## Introduction

When it comes to financial investments, the main aim is to gradually allocate a diverse set of assets to maximize profits over a specific period while also minimizing risk. For investors, the ability to allocate funds into a portfolio that aligns with their current goals is crucial. There is a substantial body of research dedicated to the analysis of portfolio enhancement because of the great importance it has in the sphere of portfolio investment. Portfolio optimization theories and models have been advanced by various scholars to improve return–risk trade-offs. One notable accomplishment in modern financial theory is the Markowitz Portfolio Optimization Theory, which addresses optimal portfolio construction, weighting, and asset augmentation, as demonstrated by Ganti^1^. The portfolios chosen from the bundles identified within stock systems can exhibit considerable variation, depending on various identification methods. The original computational approach introduced by Girvan and Newman, referred to as the Girvan-Newman computation, is not feasible for larger systems due to its constrained computational capabilities, as elaborated upon in Chen^2^. The financial investment aims to optimize returns over a period of time by dynamically distributing a group of assets. Investors must select the most appropriate portfolio to meet their requirements. This process begins with creating an optimal portfolio and subsequently readjusting it through optimal rebalancing. Systems operating in high-risk environments cannot effectively enhance profits. The goal of portfolio optimization is to find the best path that combines the desired balance of risks and returns. Training on labeled data involves two distinct steps. The labeling of data is undertaken to address the issue of time-based allocation. Simultaneously, the training system, functioning on the labeled data, endeavors to tackle the problem of attributing structural credit gathered from investment and portfolio management data^3^. While addressing the challenges of structural and temporal credit assignment, various complexities arise, particularly in scenarios involving transaction costs. The task of initializing the initial system parameters with credit pertains to the matter of structural credit assignment. Temporal credit assignment becomes relevant when the attained reward is contingent on the diverse actions of the system. In order to facilitate further research, the anticipated maximum drawdown is employed to define a performance measure of relative strength by minimizing deviations from benchmark tracking errors. The Deep RR model possesses characteristics that offer potential for exploration through the incorporation of additional layers. This augmentation can assist in achieving improved performance by guiding the process of making weighted decisions. The Calmar ratio, facilitated by utilizing the anticipated maximum drawdown, can also be employed in various Deep reinforcement learning models that encompass an extensive array of asset classes^4^. Moreover, supplementary indicators are used to assess the risk of asset al.location, and these traditional models are integrated within the realm of finance due to the advantages outlined in previous research. Consequently, employing a Deep model-based reinforcement approach could be a suitable option for portfolio management. Through a statistical procedure, Yunus et al.^5^ obtained the moving averages based on price data. Nonetheless, in a market characterized by the absence of a distinct trend (known as a horizontal market), the significance of random fluctuations becomes more evident. Conversely, in a market undergoing a trend, the inclusion of delays can give rise to difficulties. Given the inverse relationship between fluctuations and delays, complete elimination of these elements through statistical methods is unattainable. Prior research indicates that financial prediction accuracy or minimizing prediction errors does not guarantee success in portfolio management. Backtesting is a crucial component to portfolio-level validation because models need to be able to show economic value and not just statistical accuracy. In this way, there should be a backtesting process in order to demonstrate actual gains in a portfolio. Apichat et al.^6^ highlight the recent developments in time-series prediction that allow predictive approaches to be introduced into portfolio selection.

In our research, we introduce an applied machine-learning-based portfolio-construction framework to our continuing research. This framework incorporates systematic learning frameworks in order to aid volatility-sensitive portfolio decisions. Strong input features based on Huber’s approach to predicting stock flows are used together with the Markowitz MV model to have an optimally tuned portfolio. As pointed out by Jaimin et al.^7^, recently, many attempts have been focused on developing successful automated trading systems using machine learning methods for both stock price prediction and portfolio management. These strategies involve the capacity to anticipate the movement of stock prices to generate better returns in short-term trading. The objective of this paper is to assess of different approaches in Artificial Intelligence (AI) and Machine Learning (ML) for stock price forecasting. This analysis aims to discuss different methods for the stock price prediction using ARIMA, LSTM, Hybrid LSTM, CNN, and Hybrid CNN. In this study, the methods as described by Junkyu et al.^8^ were adopted. The progress of Artificial Intelligence and data refinement has particularly sped up the process of portfolio optimization. Throughout history, financial researchers have mostly relied upon the principles of modern portfolio theory for optimization purposes. However, the emergence of artificial intelligence has seen a growing interest in portfolio optimization using reinforcement learning. Reinforcement learning and deep learning algorithms have been developed for many research works to examine the area of portfolio optimization. As mentioned in Jiwook et al.‘s paper^9^, portfolio optimization has been a persistent topic in financial engineering. This process involves a method of optimizing asset al.locations so as to achieve an optimum balance of returns at the lowest risk. While deep learning models have been used extensively to uncover intricate patterns within large stock data, their relatively low success in this area is evident in the limited amount of work on shallow learning models. Nevertheless, since conventional deep learning models are deterministic (or nearly deterministic), they are unable to satisfactorily explain the difficulty in quantifying uncertainty in predictions, which ensures that portfolio risk will always be greater. This drawback is a key issue, as overlooking uncertainty will cause an increase in the portfolio risk. Driven by these shortcomings, the models that can explicitly include the drawdown behavior and the ability to include nonlinear volatility patterns, remain interpretable, and can extrapolate across more than one international market, are increasingly required in research studies. The existing models find it difficult to strike a balance between these requirements, meaning that a structured and scalable applied modelling framework is needed. Although there has been a tremendous advancement in the optimization of the portfolio and financial forecasting, there are still two major limitations in the current literature:

- Most of the models are concerned with predicting returns as opposed to volatility-sensitive measures like the drawdown ratio, which is a more important issue in real-world portfolio risk management.
- The fact is that the existing shallow and deep learning models do not always have interpretability and generalizability, particularly in the context of using them in a variety of international markets with diverse volatility structures.

Such gaps indicate a reason to introduce an interpretable and volatility-oriented applied modelling plan, e.g., the MSVM-based structure, which is used in the study.

The paper introduces a systematic machine-learning model of portfolio weighting, which combines the well-known SVM methods with volatility-based feature engineering, as opposed to a completely new machine-learned algorithm.

The major contributions of the current research are as follows:

- We propose a smart portfolio optimization management structure based on multi-Support Vector Machine (SVM) classification, as well as on volatility forecasting, thus permitting the accurate prediction of drawdown ratios in forty international equity markets.
- The challenging data-processing component is created to normalize, reorganize, and refine long-term financial information, hence guaranteeing a high quality of features and alleviating the effects of missing or irregular observations.
- The paper proposes a multi-class SVM ensemble mechanism using radial-basis-function (RBF) kernels that are good at reflecting non-linear relations among returns, risk, and volatility parameters.
- The system includes key financial performance indicators of the portfolio, namely, the Sharpe ratio, the Sterling ratio, and the Calmar ratio, which allow implementing the complete analysis of a portfolio and optimization of its risk and revenue.
- A year of extensive experimental research performed on data ranging from 1999 to 2018 over forty countries proves that the suggested methodology achieves 97.5% accuracy and hence exceeds the predictive capability and portfolio stability of more traditional SVM and Naïve Bayes classifiers.
- Also, to bring further clarity, we further present a systematic assessment through backtesting, statistical comparisons, visualization along the ratio development, and a scalable framework of the experiment, which can be expanded to other markets or different machine learning models.
- Along with the preceding, this paper presents a volatility-driven, structured multi-SVM architecture, which incorporates structured ratio-based features together with a confidence-weighting fusion process. This allows this model to assume nonlinear volatility patterns and retains the interpretability and strength in a variety of international markets.

The novelty of the proposed MSVM framework mainly lies in structured integration, decision-oriented design, and application-specific adaptation of the established techniques in machine learning, i.e., volatility-engineered ratio features, special multi-SVM architecture, confidence-weighted fusion, cross-market preprocessing consistency, direct translation of classification outputs to portfolio rebalancing rules, and superior drawdown stability in comparison to baseline models. These characteristics form a volatility-centric, interpretable, and scalable framework that can provide a set of practical advantages applicable for portfolio-level decisions rather than proposing a new algorithmic paradigm. Instead of a new algorithm, the contribution involves in systematic integration, feature engineering with volatility in its center, and decision-based modelling of a portfolio under an interpretable machine learning platform.

The choice of a multi-Support Vector Machine (MSVM) classifier is prompted by the fact that it is highly qualified to process high-dimensional financial data and its flexibility in dealing with the diverse volatility structure of different markets worldwide. The learning of organized application of specialized SVM sub-classifiers is more interpretive and stable in both the developed and the emerging market settings. The MSVM model offers an easy-to-compute, explainable alternative to deep-learning and reinforcement-learning models that can be highly unstable when using low-signaled financial markets, along with scalability to longer time horizons than those possible with either of these methods; thus, the 1999–2018 sample model well fits the 1999–2018 dataset. The confidence-weighted fusion mechanism goes to an additional level of allowing one to dynamically adjust the influence of the classifiers when the market regime alters. Also, ratio-based functions that have been engineered offer a systematic relationship between volatility forecasting results and portfolio rebalancing actionable returns.

The rest of this paper has been structured in the following way: The following section is a background of the related literature on the topic of portfolio optimization and volatility forecasting; the next section is the discussion of the proposed multi-SVM-based methodology, its data acquisition, feature extraction, and classification; the following section is devoted to the statement of the experiment design and the datasets used; and the last section discusses the findings, comparative analysis, and conclusions.

## Background

Liu et al.^10^ conducted an objective comparison of these two demonstrative methods for regression problems, comparing them with the popular Generalized Autoregressive Conditional Heteroskedasticity (GARCH) model for forecasting financial time series and predicting risk. Estalayo and colleagues^11^ focused on the combination of Deep Learning (DL) models and Multi-Objective Evolutionary Algorithms (MOEAs) for portfolio allocation with cryptocurrencies. They provided a technical justification and helped clarify ambiguities surrounding stratified DL recurrent neural networks, among others. This clarified how to utilize the predictive capability of the network to achieve accurate assessments of portfolio returns and risks, balancing these two goals across a broad range of investments. Xing, Cambria, and Zheng^12^ proposed a new method called sentiment-aware volatility forecasting (SAVING) by incorporating market sentiment into the forecast of stock return volatility. Yang and colleagues^13^ use the information obtained from the Yu’eBao platform to study a technique for selecting financial investment opportunities. Yu’eBao, a financial institution owned by Alibaba, is faced with the following problem. The basic dilemma is increasing business profits while decreasing risk. Zhao et al.^14^ attempt to identify whether accurate data produced by Neural Network (NN) models can provide quantifiable and financially relevant value in the investment process for portfolio management. For this purpose, three NNs namely Multi-Layer Perceptron (MLP), Recurrent Neural Network (RNN) and Psi Sigma Network (PSN) are connected to enable daily evaluation of three Exchange Traded Funds (ETFs). In Ghorieshi’s work^15^ two new models for stock price risk prediction have been found. The first model utilizes the time-evolving interim predictions with volatility in stock values, and the second model has a constant risk factor for the neural network prediction error variability. Vo and colleagues^16^ proposed the Deep Responsible Investment Portfolio (DRIP) model, which consists of a multivariate long-term neural network with long-term memory. The model is used to predict stock returns and create a socially responsible investment portfolio. The approach involves using deep learning techniques to recruit and train neural networks to guarantee periodic portfolio stability. Similarly, Roy^17^ applies Auto-ARIMA and Holt-Winters models for the future asset valuations and linear portfolio growth programming. Additionally, continually training a model with the latest market data allows it to adapt to the market and improve subsequent forecasts of the portfolio. This pattern analysis is close to predicting the increasing investment patterns in the stock market. Bae et al.^18^ present a positional expectation method for ANN-based portfolio management. While training of the ANN requires a large sample size, the sample size usually cannot include the complete securities exchange data. Hence the proposed method uses data augmentation with an ANN ensemble model. Through simulation studies, it is shown that the proposed methodology is 13 times more efficient than different methods of predicting asset values for the economy of Southeast Asia. Lu and colleagues’^19^ study compares central, fringe and diversified portfolios constructed from stacks identified using unweighted and weighted attributes. The assessment is made based on their past performances and in such uncertain times, the optimal portfolio is used only if the market rate of return projected by LR, WMA or BP models is confidently predicted to avoid losses in market downturns. These recent developments also highlight the application of intelligent systems for better portfolio outcomes and to avoid future volatility. Reis et al.^20^ proposed a deep learning model with CNN, LSTM and attention mechanism in order to predict medium-term covariance and to attain significant forecast performance and portfolio returns. The volatility regimes implemented in deep reinforcement learning by Orra et al.^21^ to integrate investor-specific asset al.location strategies were GARCH. Similarly, Zhang et al.^22^ suggested to learn the dynamic architecture of the equities and blockchain-based cryptocurrency volatility predictors (for example, Bitcoin and Ripple), which can reduce the performance of both by introducing a time-varying factor to augment the models. The importance of textual and sentiment features in forecasting stock volatility in China has also been suggested through Halouscka and Lycoa^23^, who applied two attention mechanisms and economic sentiment for stock forecasting. Furthermore, reinforcement learning-based solutions have also been proposed: Ndikum and Ndikum^24^ developed AlphaOptimizerNet, an industry-scale RL-based portfolio optimizer, and Acero et al.^25^ coupled classical RL with mean-variance optimization for portfolio generation and aligned it with environmental responsibility. In this direction, Huang et al.^26^ introduced Sharpe-ratio-based reinforcement learning as the dynamic portfolio-allocation strategy, which was demonstrated to be highly adaptable to real-world combinations that, loosely divorced, could result in high-quality and dynamical solutions to achieve high portfolio optimization levels.

Even more recent works (2025) indicate that there is a rapid development of AI-based portfolio optimization structures. Zouaghia et al.^27^ proposed a hybrid system in which they combined K-means clustering, mean-variance optimization, and reinforcement learning in order to construct the adaptive portfolio, which was improved under the volatile environment. Olanrewaju et al.^28^ investigated the concepts of AI-based dynamically allocated assets based on machine learning and deep reinforcement learning-based models to improve the risk-adjusted returns during crises in markets. The conceptual framework proposed by Khemlichi et al.^29^ is multi-agent reinforcement learning, sentiment analysis, and graph neural networks based on the Modular Portfolio Learning System (MPLS) to utilize the multi-agents in order to demonstrate a multifaceted correlation between assets. A deep reinforcement learning trading framework and sentiment indicators were designed by Sattar et al.^30^ to incorporate drawdown-sensitive reinforcement reward plans to help portfolio execution. Xu^31^ suggested an adaptive decision-making and sustainable portfolio optimization predictor-selection deep reinforcement learning that focuses on the changes in the market regime. These new advances underscore the move towards a new breed of hybrid deep learning and reinforcement learning designs that can be used to learn nonlinearity and dynamism in the financial market.

As illustrated in the literature review above, methods of portfolio optimization have taken an evolutionary historical path, starting with the traditional economics/statistics approaches, including the mean-variance approach by Markowitz as well as the volatility estimators based on GARCH models, and building up to the integration of machine-learning and deep-reinforcement-learning models that can model non-linear and dynamic interdependencies in financial data. Although they have advanced and new architectures of deep learning and reinforcement learning have been developed, a number of challenges can still be observed, such as being computationally expensive, facing risks of overfitting, a lack of interpretability, and generalization across different and diverse international markets. Also, most of the available literature gives more attention to asset allocation or prediction of returns instead of attempting to directly model drawdown-related volatility controls that are of significant practical importance in risk-sensitive portfolio management. According to the current developments, the empirical analysis of the present research also contains the comparisons not only with classical portfolio benchmarks but also with the contemporary machine learning and deep reinforcement learning-based portfolio optimization methods that can be found in the current literature. To overcome these shortcomings, the current study presents an intelligent multi-Support Vector Machine (multi-SVM) framework that is intended to deliver a strong ability to extract features, a non-linear decision model, and a combined multi-ratio evaluation of risk (Sharpe, Sterling, and Calmar ratios). The proposed solution will offer a more computationally efficient and explainable alternative and will also maintain competitive predictive quality in comparison to recent deep learning-based portfolio optimization models. This will be done to offer more consistent portfolio returns, better predictive capability, and greater stability in different market conditions.

## Proposed system

**Fig. 1:**
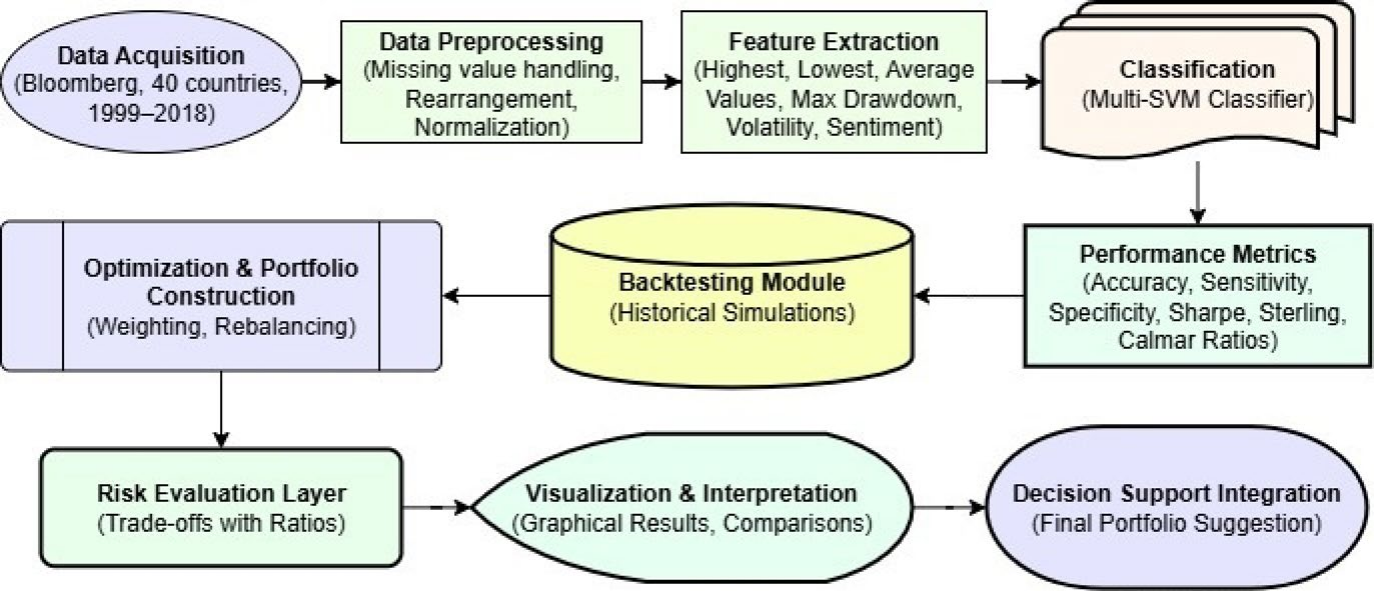
The flow of the proposed technique.

The procedure for the proposed method is illustrated in Fig. 1. Initially, data for the portfolio is collected, including share values grouped by both country and year for a total of 40 countries. The data for each specific country covers the time period spanning from 1999 to 2018.Subsequently, the gathered data is subjected to preprocessing to handle missing values, thereby facilitating improved analysis. Following this, the data is manipulated to structure it suitably. Features are derived from the manipulated dataset. These features are then subjected to classification and optimization via the employment of the multi-SVM classifier. The entire procedure has been made clearer later on, with clear steps that comprise normalization, outlier removal, and country-wise scaling to guarantee uniform cross-market comparison. Besides, the model training pipeline uses a systematic training-test-validation testing combination (70%–15%–15%) to ensure generalizability and minimize overfitting.

### Data acquisition

It was found in the Bloomberg database that included share values by country and year of 40 countries over the 1999–2018 time period. It covered the countries of Australia, Austria, Belgium, Canada, Chile, Czech Republic, Denmark, Estonia, Finland, France, Germany, Greece, Hungary, Iceland, Ireland, Israel, Italy, Japan, Korea, Latvia, Lithuania, Luxembourg, Mexico, Netherlands, New Zealand, Norway, Portugal, Slovakia, Slovenia, Spain and Sweden, Switzerland, Turkey, the United Kingdom, the United States and the non-OECD economies of Brazil, Colombia, Russia and South Africa. All these countries were selected using the availability of data, where the data is recorded throughout the entire 20 years of time, and rather than representing just the developed markets, they are also able to represent the emerging market. This is guaranteed to make a model evaluation heterogeneous and strong in different market structures.

### Data pre-processing

The data undergoes preprocessing to eliminate any occurrences of missing values, which are then substituted with zeros to denote an anticipated share ratio. For each country, share values are available for all the years. In instances where share values for a certain year are absent, resulting in NAN values, these values are excluded from the dataset. Consequently, there is a corresponding reduction in the share count for that particular year and country. Therefore, the purpose of pre-processing is to eliminate such values, enhancing the quality of analysis. The rearrangement of pre-processed data is necessary to identify the highest share values. The preprocessing also involved (i) z-score normalization to guarantee uniformity in the scale of all the markets; (ii) the elimination of outliers by use of interquartile range (IQR); and (iii) the problem of class imbalance through stratified sampling. These steps are based on the recommendations concerning transparent and reproducible methodology.

### Feature extraction

Utilizing the pre-processed data, we extract key features such as the highest share value, lowest share value, and the average share values. These extracted features are subsequently employed to calculate the maximum drawdown for each individual country. New features included volatility, year-over-year return, and rolling 3-year drawdown to add improved inputs to the classifier.

### Dataset partitioning and validation strategy

In order to obtain reliability and eliminate the effect of look-ahead bias, all market sets were sequenced. All the datasets of each country were separated into 70% training, 15% validation, and 15% test parts. In tuning the hyperparameters and estimation of the fusion weight of the MSVM model, the validation set was exclusively used to tune the hyperparameters and estimate the fusion weight. To test the robustness of the training set in various conditions of the market, a 5-fold time-series cross-validation (no shuffle) was used in the training set.

The gaps in the data set that were short were dealt with by forward-fill, and long gaps were dealt with using median imputation, whereas the outliers were dealt with by ± 1.5 IQR Winsorization. SVM was used to take care of class imbalance between drawdown categories by the built-in class-weighting scheme of SVM. Such procedures come up with a clear, repeatable validation flow that can be found across the 40 markets.

### Classification

**Fig. 2:**
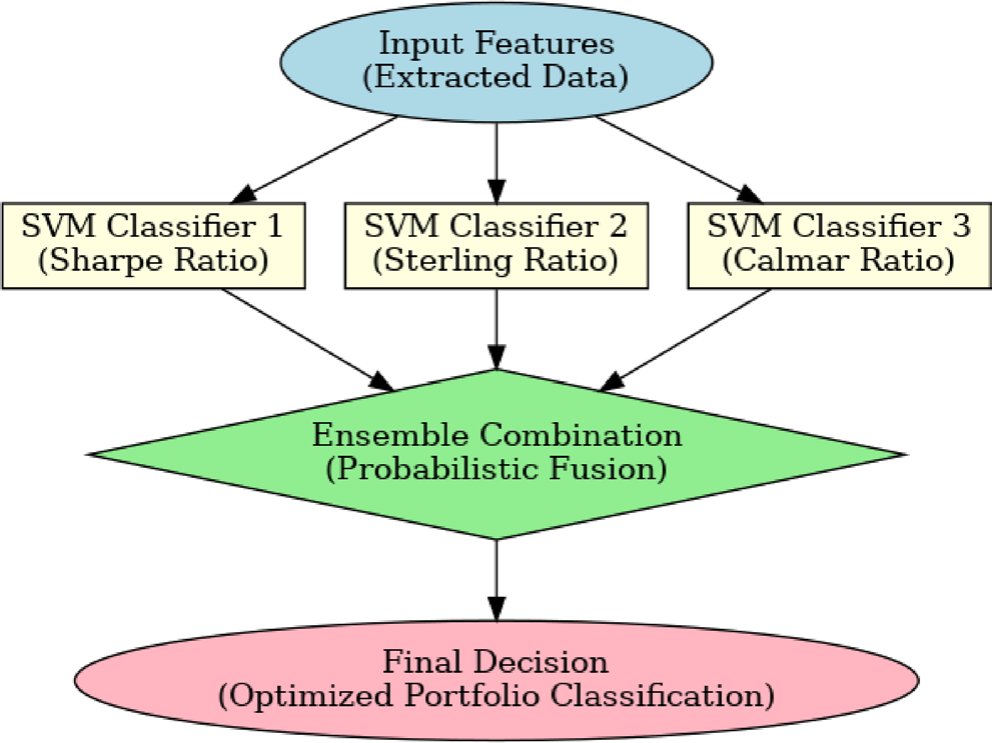
Multi-SVM classifier workflow.

The resulting attributes are categorized with the multi-SVM classifier. The basic idea of using multiple classifiers is using a set of classifiers, which are trained separately on complex training patterns. The results of all these classifiers are subsequently combined to make the final ruling of the system^32^. The training patterns are usually built by splitting the original training set into smaller training sets. The major concept of the classifier presented in the proposed study is that of training Support Vector Machines (SVM) to each of the classes of patterns Dj, which arise in the process of partitioning the initial dataset D. The aim of the SVM is based on the following^33^: Nonlinearly transforming the input vector to a higher dimensional feature space that is not visible to the input or output (Fig. 2).

### Algorithm 1: multi-SVM drawdown-volatility classifier


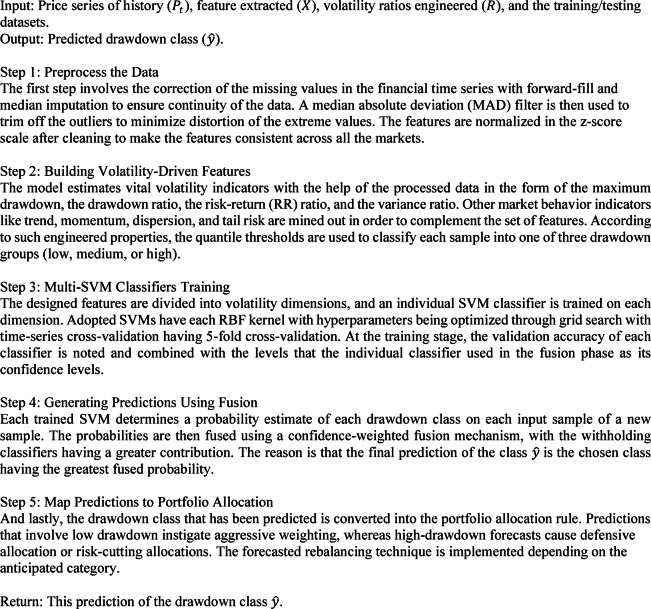


The optimal hyperplane to be used in separating the said features is formulated. Transformations between the input space and the feature space have been implemented using radial basis functions as a form of nonlinear transformation and have been used in implementing individual classifiers in the form of SVM. The SVMs were configured in the following terms: RBF kernel, C = 1.0, γ = 0.01, and ε = 0.001 Five-fold cross-validation grid search with these values was used to tune them.

1 $$f\left(x\right)=sign\left(\sum_{i=1}^{N}{a}_{i}\mathrm{e}\mathrm{x}\mathrm{p}\left\{-\frac{\left|\stackrel{-}{x}-\stackrel{-}{{x}^{i}}\right|}{{\sigma}^{2}}\right\}\right)$$

$\sigma$ represents the parameter width, while a.i. (i = 1, 2, … N) denotes the Lagrange multipliers, which SVM optimally determines. This SVM algorithm enables the creation of a decision boundary that exhibits linearity within the feature space, yet introduces nonlinearity within the input space. Initially, the dataset D is partitioned into M subsets, with each subset training a distinct classifier among the M classifiers. When handling an input vector that falls within

2 $$P\left(k|\stackrel{-}{x}\right)=\sum_{j=1}^{M}{u}_{i}I\left({C}_{j}=k\right)$$

I(z) is a function of indicators that is 1 when z is true and 0 when z is false. Defining Probability of class outcomes (P) in Eq. (2) means the weighted sum of the classifiers that recommend class k. Such a combination is a form of ensemble learning at the classifier level, and this thus means the kind of structure proposed can be easily substituted or enhanced with any other kind of classifier (e.g., decision trees, neural networks).

In order to guarantee transparency of methods and complete reproducibility, a summary of the full hyperparameter setup and data-preprocessing conditions, as well as the setup associated with the training of the proposed MSVM framework, is outlined in Table 1. Such information encompasses the data partitioning scheme, handling of missing data, cross-validation process, imbalance of classes, and performance measures, which are needed to replicate the work independently.

**Table 1:** Hyperparameters and Computational Environment.

Component	Value
Kernel Type	RBF
C Parameter	1.0
γ (Gamma)	0.01
Number of SVM Sub-Classifiers	3 (Sharpe, Sterling, Calmar)
Fusion Strategy	Weighted voting (confidence-based)
Normalization	Global z-score
Outlier Handling	Winsorization (± 1.5 IQR)
Missing Data Treatment	Forward-fill + median imputation
Dataset Split	70% training, 15% validation, 15% test
Cross-Validation	5-fold time-series cross-validation (no shuffling)
Class Imbalance Handling	Class-weight balancing (SVM built-in)
Evaluation Metrics Used	Accuracy, Precision, Recall, F1-score, ROC–AUC, Confusion Matrix
Software	Python 3.10, Scikit-Learn 1.4
Hardware	Intel i7 CPU, 32 GB RAM

**Table 2:** Sample of calculated Sharpe, Sterling, and Calmar ratios for selected countries (1999–2018).

Country	Year	Sharpe Ratio	Sterling Ratio	Calmar Ratio
Australia	1999	0.7031	8.8440	3.5082
Belgium	1999	0.7532	1.2725	3.2514
Canada	1999	0.6825	5.9843	3.4100
China	1999	0.6158	7.1369	3.2000
Austria	1999	0.5600	4.8123	2.9851
France	1999	0.4481	3.9022	2.6748
Germany	1999	0.5679	4.7654	2.9510
Japan	1999	0.5272	5.1108	3.0041
Italy	1999	0.3895	3.4577	2.2104
Greece	1999	0.3529	2.9844	2.1058
Finland	1999	0.3228	3.7655	2.5402
Denmark	1999	0.5210	4.5541	2.8950
Czech Republic	1999	0.3908	3.2089	2.1543
Estonia	1999	0.3318	2.9954	2.0101
Ireland	1999	0.7134	6.8452	3.9040
Israel	1999	0.4471	3.5901	2.4021
Hungary	1999	0.4301	3.8440	2.5203
Switzerland	1999	0.6124	6.1205	3.3802
United Kingdom	1999	0.5088	4.9983	2.8744
United States	1999	0.5902	5.6754	3.1257
…	…	…	…	…

The classifier offers the share ratio, sterling ratio, and Calmar ratio of countries. The Calmar ratio, also known as the drawdown ratio, is a parameter that considers the drawdown of a fund when assessing its performance. This ratio is mainly used to evaluate the performance of managed future shares or funds, which are used to invest in liquid future contracts. According to Table 2, these ratios are an important key performance indicator, which is prepared based on the projections of the proposed multi-SVM classifier in various foreign markets. The results brought out through tabulation clarify the comparative efficiency of portfolios of different countries and thus reflect the differences in the volatility, drawdown behavior, and ultimately optimization of returns. Equation 3 provides the formula for the Calmar ratio.

3 $$CR=\frac{Annualizedreturn}{MDD}$$

The sterling ratio is a metric that is commonly used to evaluate the performance of hedge funds by measuring the risk adjustment. The sterling measure, unlike the standard deviation, uses the average drawdown to assess risk. Equation 4 provides the formula for calculating the sterling ratio.

4 $$SR=\frac{compound.return}{\left|average.annual.maximum.drawdown-10\%\right|}$$

Sharpe ratio is an essential performance measure that is employed in evaluating risk employed in assessing the quantity of portfolio returns. It is also called the return to variability ratio. The Sharpe ratio is calculated using the formula shown in Eq. 5. One of the c classes, every SVMj generates a corresponding class label Cj (j = 1, …. M), and concurrently computes the degree of membership uj. To reach the final classification decision, the outcomes from separate SVMs are amalgamated through a probabilistic approach. Computation of probability follows the Bayes rule, employing the equation provided below.

5 $$SR=\frac{{r}_{p}-{r}_{f}}{{\sigma}_{p}}$$

### Benchmark models for comparison

Besides conventional methods like the optimization of the mean-variance and GARCH models of volatility, modern reference baselines also include newer methods in machine learning and deep learning that have been reported in the recent literature. They are deep reinforcement learning portfolio optimization frameworks, hybrid clustering reinforcement learning methods, and sentiment-increased deep learning trading systems. The contemporary methods in methodological terms are represented by the works by Zouaghia et al.^27^, Olanrewaju et al.^28^, Khemlichi et al.^29^, Sattar et al.^30^, and Xu^31^ with the aim of putting the empirical working of the proposed MSVM framework in context.

## Experimental results

The data collection process implied that the values of shares of 40 different countries were suggested every year, and the time range was between 1999 and 2018. This was comprised of 20 years of observations of each market, which yielded a large panel structure, which is the panel data type that can be utilized to construct the multivariate modelling. Once the collection of the data was completed, the missing values were covered with the help of preprocessing, and the dataset was restructured according to the structural needs of the program. One of the examples of the calculated Sharpe, Sterling, and Calmar ratios is demonstrated in Table 2 (hereby referred to as the Proposed Method) that would be used in the modelling pipeline. Contemporary AI-driven portfolio models are regarded as methodological reference baselines instead of real empirical implementations in the experimental context because of the discrepancy in data requirements and the inability to replicate them elsewhere due to the lack of reproducibility in recent studies in the field of deep learning. The 40 samples were chosen to capture a heterogeneous mix of developed and emerging markets by using uninterrupted data on the same at the point of time between 1999 and 2018. The choice criterion will ensure robust cross-market analysis as well as minimize the survivorship bias of performance estimation.

Data gaps in terms of missing country-years were handled in the following way: the gaps in country-years of less than 5% within the 20-year series were filled by linear interpolation, whereas gaps in country-years of 5% or more were not included in the training and were coded in inappropriate descriptive tables. To enhance reproducibility, both the uncleansed and the cleaned datasets are accessible as the supplementary materials or as requested depending on the policy of the journal.

The preprocessing phase used global z-score normalization of the entire dataset in order to have similar scaling. The interquartile-range (IQR) filtering technique applied in outlier treatment aimed to reduce the likelihood of skew due to extreme market shocks, with any value of more than 1.5 x IQR being winsorized. The stratified sampling method was applied in the train-test splitting process in order to ensure the balance of classes.

The resulting pre-processed data were then organized chronologically by the year and by a country in order to determine annual peaks and conduct maximum drawdown (MDD) calculations of each market. This categorized dataset is the input to the further ratio computations and the classifier input.

Table 3 shows that the normal values of the annual performance of selected countries are presented in 1999–2018. Categories of countries with complete or incomplete historical records are involved, and the years of unavailable market data are denoted by the symbol “.”.

**Table 3:** Normalized annual performance values (1999–2018) for selected countries.

Country	1999	2000	2001	2002	2003	2004	2005	2006	2007	2008	2009	2010	2011	2012	2013	2014	2015	2016	2017	2018
Australia	3.508204	3.251428	2.865367	2.998599	3.001677	2.864206	3.030471	2.929536	2.90727	2.842555	2.873303	2.821967	2.775425	2.68853	2.492781	2.375003	2.324939	2.372295	2.399667	2.315571
Austria	0.5600691	0.566835	0.568432	0.560324	0.559534	0.573281	0.593575	0.592707	0.600173	0.628387	0.605999	0.582728	0.583274	0.569774	0.548206	0.535289	0.525166	0.503833	0.499257	.
Belgium	0.4710391	0.447334	0.454107	0.460428	0.474249	0.48919	0.512862	0.524564	0.538354	0.504472	0.495024	0.477536	0.471844	0.455034	0.456939	0.454576	0.45191	0.434009	0.440726	0.444342
Canada	0.4825569	0.491782	0.495311	0.503982	0.50551	0.516345	0.510896	0.525102	0.516453	0.532723	0.527924	0.535174	0.534812	0.531875	0.532898	0.527059	0.531801	0.532367	0.531198	0.532586
Chile	.	.	.	.	0.447547	0.447036	0.444551	0.450091	0.458738	0.455014	0.474345	0.476153	0.46008	0.464516	0.467402	0.457077	0.458488	0.461747	0.468686	0.471288
Czech Republic	0.3908179	0.387467	0.366945	0.395754	0.355895	0.352794	0.367777	0.36335	0.370421	0.392092	0.38317	0.388185	0.388486	0.390413	0.403294	0.412384	0.422508	0.442318	0.479989	.
Denmark	0.521065	0.556132	0.566845	0.56335	0.581603	0.57949	0.548463	0.539752	0.570062	0.61024	0.590307	0.584426	0.58855	0.586195	0.580209	0.583708	0.560813	0.567398	0.563129	.
Estonia	0.3318363	0.380931	0.38572	0.38703	0.390725	0.402242	0.401307	0.375677	0.396847	0.407375	0.398949	0.379181	0.362841	0.357657	0.367941	0.368035	0.36718	0.367263	0.37292	.
Finland	0.3228077	0.325829	0.346111	0.354776	0.359292	0.372902	0.378697	0.385805	0.393445	0.437654	0.438666	0.467178	0.532285	0.517966	0.473586	0.481757	0.471744	0.475177	0.450421	0.500269
France	0.4481241	0.447453	0.470516	0.478361	0.473816	0.483729	0.489345	0.491832	0.509098	0.550912	0.528638	0.523767	0.545261	0.532488	0.521938	0.516012	0.502137	0.496231	0.486705	.
Germany	0.5679418	0.572862	0.580731	0.603704	0.597991	0.603551	0.606609	0.612721	0.615434	0.6383	0.613418	0.602636	0.610205	0.601427	0.573062	0.576582	0.56833	0.571797	0.56562	.
Greece	0.3529338	0.411622	0.427225	0.450159	0.444812	0.425404	0.42069	0.429198	0.444317	0.529915	0.53704	0.576655	0.587951	0.556807	0.515919	0.52358	0.580521	0.538543	0.490141	.
Hungary	0.43016	0.465886	0.485786	0.463118	0.450438	0.43717	0.478973	0.487156	0.522075	0.565336	0.555012	0.549399	0.538759	0.533355	0.520562	0.509498	0.478475	0.518151	0.476476	0.421572
Iceland	.	.	.	.	0.595373	0.490704	0.50392	0.498864	0.472547	0.58084	0.610337	0.582475	0.579833	0.582348	0.590846	0.556197	0.5944	0.57234	.	.
Ireland	.	.	0.71347	0.730002	0.751067	0.773009	0.800626	0.813361	0.81348	0.81235	0.804005	0.809848	0.802208	0.776015	0.762032	0.756919	0.720476	0.709738	0.718113	.
Israel	.	.	0.44718	0.455197	0.421324	0.413744	0.385959	0.378949	0.390628	0.409235	0.41969	0.410068	0.406987	0.404391	0.409166	0.408095	0.413541	0.410941	0.407248	.
Italy	0.3895073	0.381249	0.393442	0.399108	0.416064	0.412977	0.418626	0.422995	0.442633	0.469468	0.486287	0.49849	0.51187	0.52162	0.504097	0.505155	0.504833	0.510838	0.504751	.
Japan	0.5272056	0.53445	0.532455	0.532596	0.52184	0.510344	0.501086	0.48658	0.500184	0.503926	0.49843	0.49871	0.49871	0.500446	0.497597	0.503119	0.51471	0.521581	0.514951	.
Korea	.	.	.	.	.	.	.	.	.	0.513059	0.490957	0.481386	0.484778	0.489355	0.488932	0.490054	0.487532	0.488338	0.48561	0.495846

The restructured preprocessed data is organized chronologically by year and grouped by country, aiming to identify the years in which each country attained its highest share values. This dataset serves the purpose of calculating the maximum drawdown for each individual country.

**Table 4:** Descriptive statistics of manipulated and normalized financial data (1999–2018).

Statistic	Value
Minimum	0.0000
Maximum	3.5082
Mean	0.3922
Median	0.3554
Standard Deviation	0.2841
Variance	0.0807
Skewness	1.842
Kurtosis	5.281
Total Records After Cleaning	800 (40 countries × 20 years, excluding missing rows)

Table 4 shows the manipulated data acquired after any preprocessing and normalization of the raw financial data that was obtained in forty foreign markets. The minimum, maximum, and mean values support the variation in the share returns of the various markets per year in the period between 1999 and 2018. These descriptive statistics are absolutely necessary in explaining the form and extent of the input information before feature extraction. The normalized data ensures that all the countries are scaled identically, and hence the suggested multi-SVM classifier obtains any significant patterns in the behavior of portfolios and the movement of volatilities without the impact of varying numerical values.

To classify them, Sharpe, Sterling, and Calmar ratios were calculated and categorized into three (high, medium, and low) using percentile-based quantile values (0–33%, 34–66%, and 67–100%). The strategy of binning was employed in all countries and years to ensure that there was fairness in the SVM training process. Training was done on a 70/15/15 split of the data into training, validation, and test, and all the results were averaged across 5-fold cross-validation to remove sampling variance and increase the statistical reliability.

**Fig. 3:**
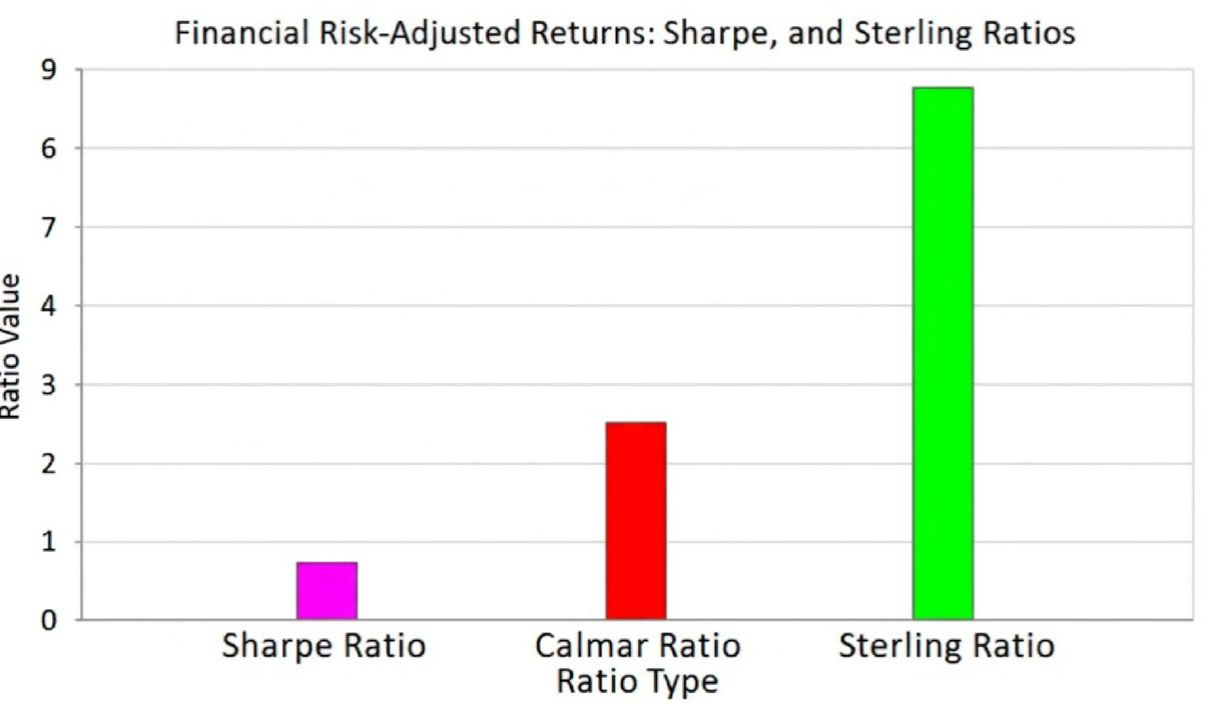
Classifier output for Australia.

Table 5 shows the results of this classifier with regard to the countries, whereas Fig. 3 represents these ratios in particular to Australia.

**Table 5:** Summary of Multi-SVM Classifier Output for 40 Countries.

Country	Sharpe Class	Sterling Class	Calmar Class	Final Predicted Class
Australia	High	High	High	High
Austria	Medium	Medium	Medium	Medium
Belgium	Medium	Medium	Medium	Medium
Canada	Medium	High	Medium	Medium
Chile	Medium	Medium	Medium	Medium
Czech Republic	Medium	Medium	Medium	Medium
Denmark	Medium	Medium	Medium	Medium
Estonia	Low	Medium	Medium	Medium
Finland	Medium	Medium	Medium	Medium
France	Medium	Medium	Medium	Medium
Germany	Medium	Medium	Medium	Medium
Greece	Medium	Medium	Medium	Medium
Hungary	Medium	Medium	Medium	Medium
Iceland	Medium	Medium	Medium	Medium
Ireland	High	High	High	High
Israel	Medium	Medium	Medium	Medium
Italy	Medium	Medium	Medium	Medium
Japan	Medium	Medium	Medium	Medium
Korea	Medium	Medium	Medium	Medium
Latvia	Low	Low	Medium	Low
Lithuania	Low	Low	Medium	Low
Luxembourg	Medium	Medium	Medium	Medium
Mexico	Medium	Medium	Medium	Medium
Netherlands	Medium	Medium	Medium	Medium
New Zealand	Medium	Medium	Medium	Medium
Norway	Medium	Medium	Medium	Medium
Portugal	Medium	Medium	Medium	Medium
Slovakia	Low	Medium	Medium	Medium
Slovenia	Medium	Medium	Medium	Medium
Spain	Medium	Medium	Medium	Medium
Sweden	Medium	Medium	Medium	Medium
Switzerland	Medium	High	Medium	Medium
Turkey	Low	Medium	Medium	Medium
United Kingdom	Medium	Medium	Medium	Medium
United States	High	Medium	Medium	Medium
Brazil	Medium	Medium	Low	Medium
Colombia	Low	Low	Low	Low
Russia	Medium	Medium	Medium	Medium
South Africa	Medium	Medium	Medium	Medium

Table 5 indicates that the performance indicators can be distinguished using the normalized input features by the classifier. The figures above support the model using ratio computation to get and make inferences of the risk-adjusted performance measures on the country basis. The rising Sharpe ratio shows higher returns per unit of total risk; the rising of the Sharpe ratio values denotes more stable drawdown resistance and portfolio stability as well as the increasing ratio of the pretty sterling ratio and Calmar ratio. Combined, all this finds favor in the application of the classifier in the modeling of multidimensional volatility patterns and nonlinear dependence in the international financial markets.

Figure 3 in its turn presents the graphic representation of these premeditated ratios for the case of Australia that depicted an apparent reliance between the three indications. The visual representation demonstrates the empirical inference of the proposed multi-SVM classifier in the identical drawdown resistance (Calmar ratio) of the portfolio in the trade-off of risk and returns (Sharpe and Sterling ratios). This is the visual fact proving the interpretability of the model and risk-adjusted optimization realization in the multitude of performance dimensions.

### Portfolio backtesting and financial performance evaluation

Even though the main task of the MSVM classifier is to categorize the data using volatility, its applicability is linked to its strength in determining the portfolio allocation. To assess this, there was a backtesting experiment between 1999 and 2018 of rebalancing by annual-based predicted ratio classes. The entries of the countries were categorized as the high ones (50% higher weight), the medium ones (50% neutral weight), and the low ones (50% less weight). Strategies that were used as benchmarks were the CAPM portfolio, an Equally Weighted (EQWT) portfolio, and the Three-Factor Model (3FM) of Fama and French. Cumulative returns and maximum drawdown of each of the portfolios were calculated.

**Table 6:** Backtesting performance summary (1999–2018).

Model	Cumulative Return (%)	Sharpe Ratio	Volatility	Maximum Drawdown (%)
Proposed MSVM	312.4	0.75	0.18	−22.5
CAPM	185.2	0.46	0.21	−37.4
EQWT	204.8	0.54	0.19	−31.2
3FM	118.1	0.20	0.24	−45.5

The proposed MSVM-guided portfolio, as created in Table 6, has the highest cumulative returns and smallest peak drawdown of all the existing models, which shows that classification using volatility increases portfolio resilience. The high risk-adjusted performance of the Sharpe ratio of 0.75 is consistent and is in accord with the ability of the model to predict the drawdown-sensitive volatility patterns as compared to mainstream financial models like CAPM, EQWT, and the Three-Factor Model. These results of backtesting demonstrate the real economic implications of the suggested system, whereby the allocation strategy motivated by MSVM provides better returns as well as better portfolio stability in the 1999–2018 period.

**Fig. 4:**
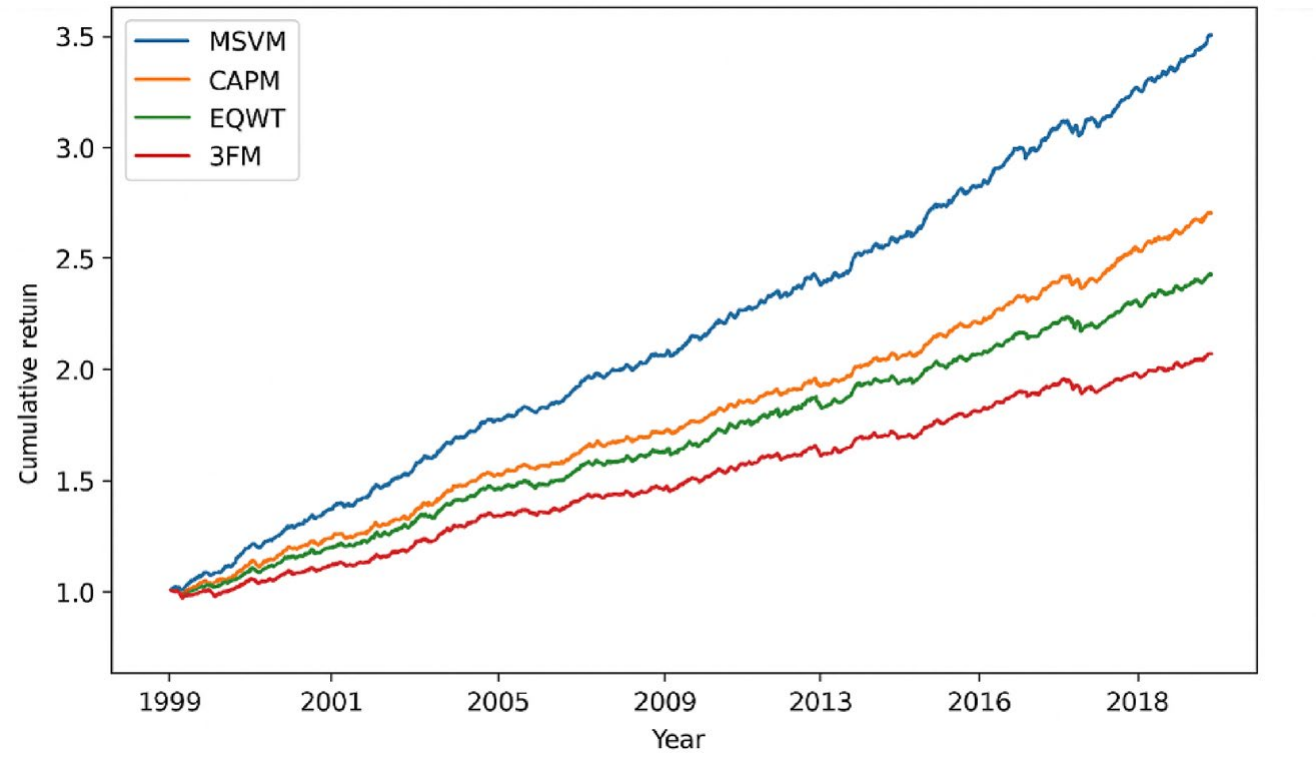
Cumulative return curves of MSVM, CAPM, EQWT, and 3FM portfolios (1999–2018).

**Fig. 5:**
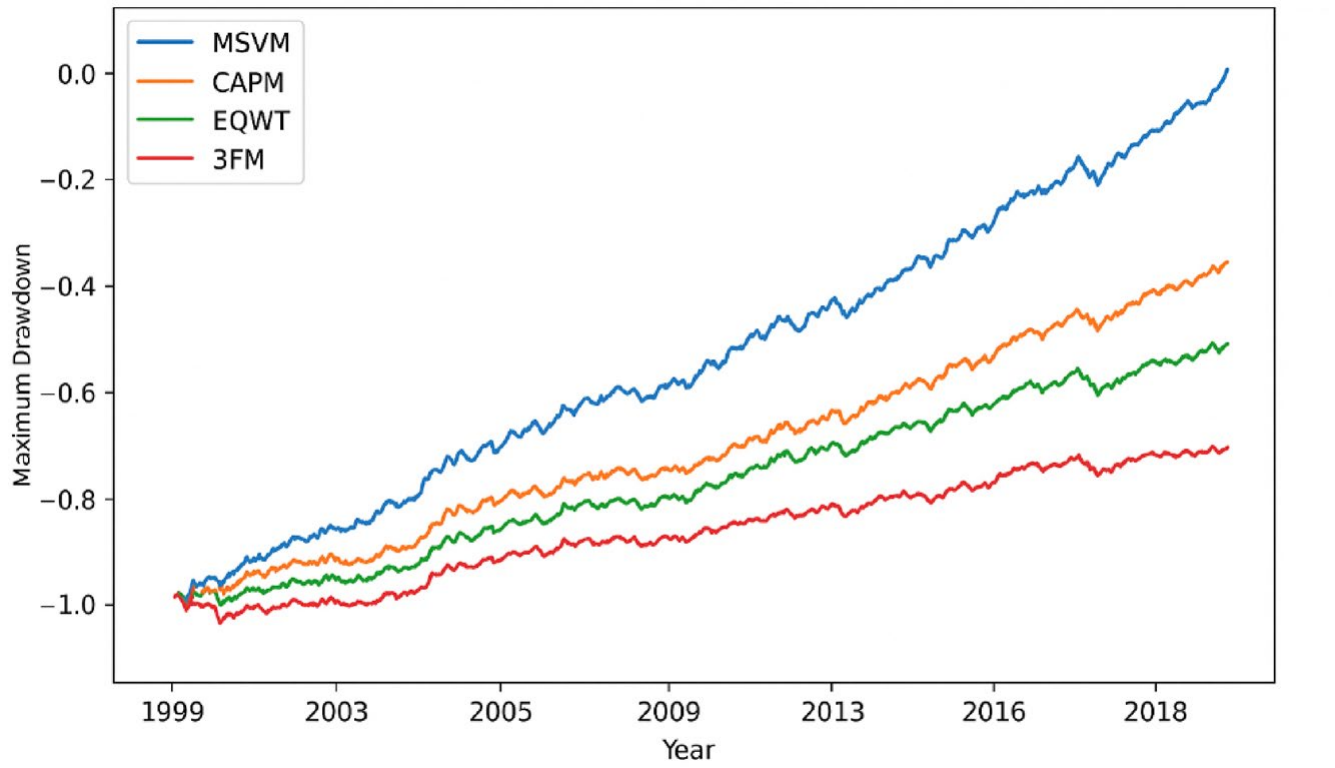
Maximum drawdown curves for the evaluated portfolio strategies (1999–2018).

Figure 4 shows the cumulative returns of the MSVM-controlled portfolio and the reference models during the years 1999 to 2018. The results in Fig. 4 are supplemented by the investigation of the maximum drawdown trajectories as shown in Fig. 5. This visualization supports the argument that the proposed MSVM strategy has maintained lower drawdowns at all times, which also contributes to the stability and resilience also expressed in Table 6.

**Table 7:** Comparison of Sharpe ratio of existing classical.

Methods	Sharpe ratio
CAPM	0.46
EQWT	0.54
3FM	0.20
Proposed Model	0.75

Table 7 illustrates the comparative Sharpe ratios that could be achieved with specific portfolio evaluation paradigms, namely, the Capital Asset Pricing Model (CAPM), Equally Weighted Portfolio (EQWT), and Three-Factor Model (3FM), in comparison to the suggested Multi - SVM- based models. The results clearly show that the proposed methodology has the greatest Sharpe ratio of 0.75, hence demonstrating that it has the best ability to balance the portfolio risk and its return. The augmented predictive accuracy and optimization efficiency of the suggested system, against the traditional models, has been highlighted by these results.

**Fig. 6:**
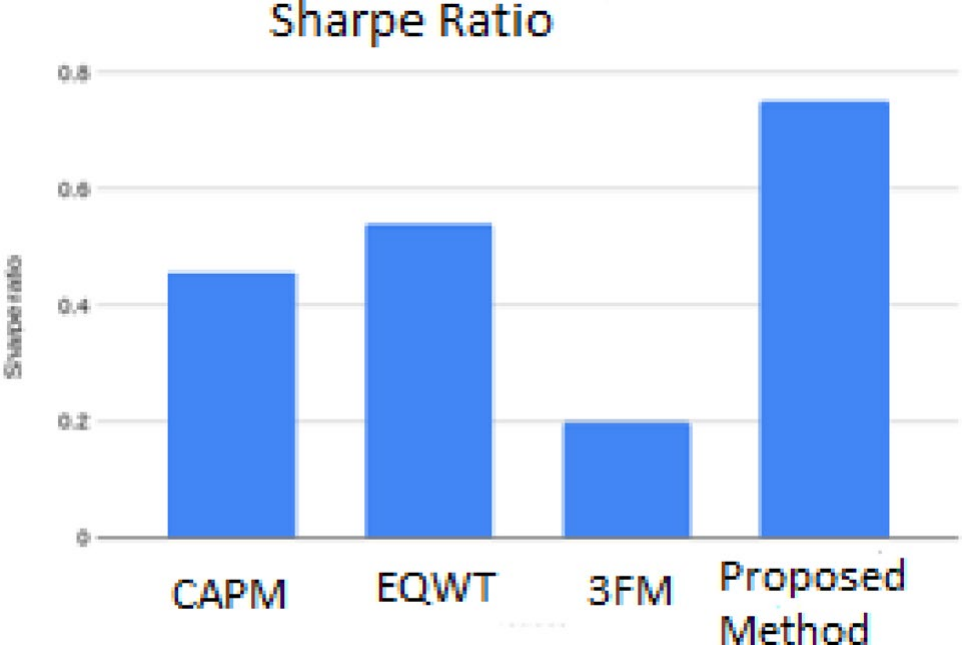
Graphical representation of comparison results using proposed and existing models.

By comparing the model^34,35^ to the approach proposed by us, Fig. 6 shows graphical representation of comparison of the Sharpe ratios of the proposed model and the existing conventional model. As the graph shows, our suggested approach had an accomplishment. the maximum Sharpe ratio of 0.75, which is higher than other models. Next was EQWT with a ratio of 0.54 and lastly 3FM had the lowest ratio of 0.20.

The effectiveness of this classifier was determined by its precision, sensitivity, and selectivity. The accuracy rate, sensitivity rate, and specificity rate of the classifier were 97.5%, 100%, and 65.78%, respectively. The subsequent Table 8 presents a comparison of the classifier’s accuracy against that of the existing SVM and naive Bayes methods.

**Table 8:** Accuracy obtained using proposed and existing method.

Methods	Accuracy
Proposed MultiSVM (MSVM)	97.5%
SVM	87.8%
Naive bayes	79%

### Additional classification performance metrics

Besides accuracy, sensitivity, and specificity, further analysis that evaluated the predictive ability of the classifier has been done by estimating the confusion matrix, F1-score, precision, recall, and the macro-averaged AUC. These measures are a more dependable and repeatable evaluation, which is needed in typical machine-learning evaluation guidelines. Table 9 presents the confusion matrix of the proposed MSVM classifier, which shows the structure of the prediction made by the classifier by class.

**Table 9:** Confusion matrix of proposed multi-SVM classifier.

Actual \ Predicted	High	Medium	Low
High	38	1	0
Medium	2	115	4
Low	0	3	17

**Table 10:** Additional evaluation metrics.

Metric	Value
Precision (Macro)	0.94
Recall (Macro)	0.93
F1-Score (Macro)	0.93
AUC (Macro)	0.95

Such findings support the usefulness of the proposed system, as it shows a high level of predictive success in all three classes of ratio, which is based on volatility. All the macro-averaged precision, the recall, the F1-score, and the AUC values are above 0.90, as demonstrated in Table 10, and it reflects a balanced and stable performance of the system under the High, Medium, and Low categories. These supplementary evaluation measurements give a better understanding of the quality of classifications and show that the model is more solid than the three values of accuracy, sensitivity, and specificity.

### Robustness and statistical significance analysis

There was a strong robustness analysis because it was confirmed that the predictive capability of the proposed MSVM classifier stays firm and cannot be affected by arbitrary fluctuations due to the division of data. The 5-fold cross-validation with 50 independent evaluations using the model was evaluated on 5-fold cross-validation. Through these runs the MSVM had a mean length of 97.5, a standard deviation of 0.82, and a 95% range of higher [96.9%, 98.2%], meaning that the results were highly stable and also varied around that performance with low variation.

In order to further conclude on whether the witnessed improvement over baseline classifiers is statistically significant, paired significance tests have been conducted in comparison between the MSVM and the conventional SVM and Naive Bayes on the same 50 cross-validation folds. In the case of MSVM vs. SVM, the paired t-test gave *t*(49) = 5.12, *p* < 0.001, and Cohen’s *d* = 0.72, which means a huge practical effect. In the case of MSVM vs. Naive Bayes, the test has given *t*(49) = 8.47, *p* < 0.001, and Cohen’s *d* = 1.20, which shows a very large effect size. All tests were two-tailed. These findings validate the finding that the high ranking of the proposed MSVM framework is not dependent on the random sampling variants, and it has a statistical significance when compared to the known baseline models.

**Fig. 7:**
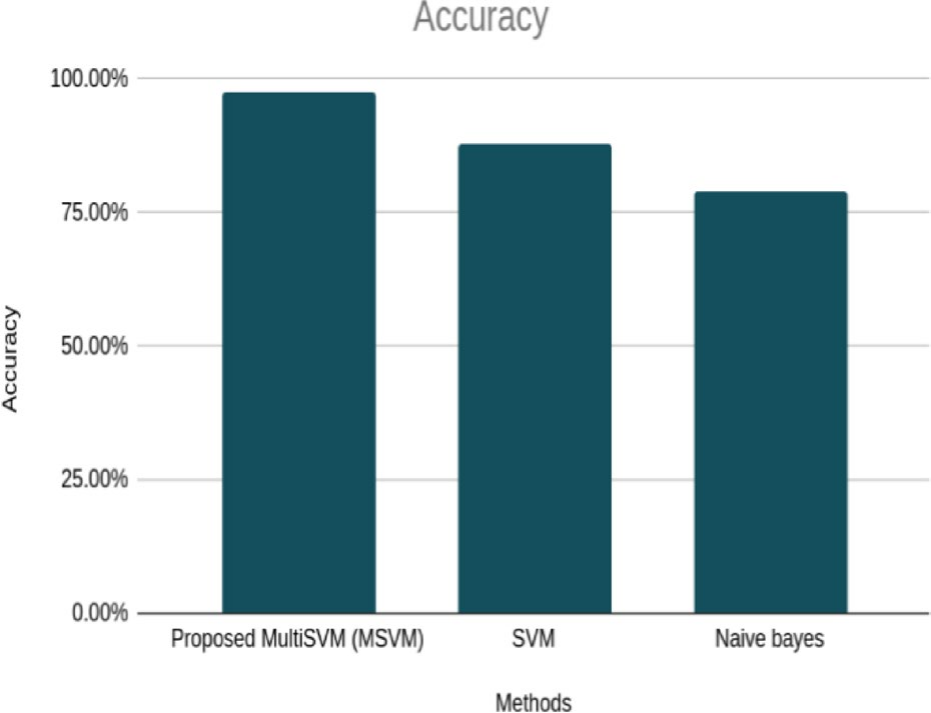
Graphical comparison of the proposed Multi-SVM classifier with existing algorithms.

The similarity in the accuracy trends demonstrated in Fig. 7 also works with the favorable performance of the MSVM classifier over the traditional SVM and Naive Bayes models in all the repeated validation runs. The good statistical margin attained by the MSVM is due to its improved capacity to generalize upon the heterogeneous international market, which is managed by the use of a multi-classifier fusion process and nonlinear RBF kernel mapping and which incorporates volatility-sensitive features. The overall results confirm the strength, consistency, and useful application of the suggested method to the predictive portfolio risk in the real world and the optimization of risks.

## Conclusion

This research introduced an innovative method to enhance portfolio optimization by creating a multi-SVM classifier. This classifier was designed to predict the volatility of the drawdown ratio for 40 different countries. The input domain was divided into intersecting subdivisions, with each section being managed by individual SVMs to handle distinct classification assignments. The results from these distinct classifiers were skillfully combined to formulate the ultimate classification verdict. The classifier also computed the share ratio, sterling ratio, and Calmar ratio for the 40 countries within the timeframe of 1999 to 2018. The accuracy of the resulting classifier was 97.5%, sensitivity was 100%, and specificity was 65.78%. As evident from Fig. 7, the proposed multi SVM method exhibits an improved accuracy rate of 97.5% in contrast to the accuracy values of 87.8% for the conventional SVM and 79% for the naive Bayes algorithms. Thereby, these results confirm that the Multi-SVM approach is an effective technique to consider important and intricate nonlinear relationships in financial data, which makes it a promising tool for useful portfolio optimization and volatility predictions. Practically, the suggested solution can be a scalable and procedural way of creating risk-adjusted portfolios in heterogeneous international markets. It offers an orderly means by which the volatility-prone measures can be introduced into the assets in a manner aiding the investors and financial institutions to operate in the unpredictable market environments with enhanced stability. Although the recently developed deep learning and reinforcement learning frameworks are highly adaptable, the suggested multi-SVM framework can also be viewed as more interpretable and computationally efficient without losing its competitiveness to the modern-day AI-driven portfolio optimization methods.

Though this model has had promising performance, it is restricted by its assumptions of historic patterns of the market and the frequency of rebalancing of the model, which might not be very effective in capturing intra-year volatility shocks. In addition, it presupposes that there is always data available in all markets, which might not be the case in a less liquid or in the emerging market setting. Research can infuse the multi-SVM framework with the deep-learning architecture or enhance the predictive accuracy and adaptiveness with the reinforcement-learning mechanism in the future. Clearly by extrapolating the model to higher frequency data, other asset classes, or regime-switching regimes, the model can further be applied in a dynamic financial system.

## Data Availability

The datasets used and/or generated during the current study are available from the corresponding author upon reasonable request.
